# Neural Origins of Human Sickness in Interoceptive Responses to Inflammation

**DOI:** 10.1016/j.biopsych.2009.03.007

**Published:** 2009-09-01

**Authors:** Neil A. Harrison, Lena Brydon, Cicely Walker, Marcus A. Gray, Andrew Steptoe, Raymond J. Dolan, Hugo D. Critchley

**Affiliations:** a Wellcome Trust, Centre for Neuroimaging, Institute of Neurology, University College London, London, United Kingdom; b Institute of Cognitive Neuroscience, University College London, London, United Kingdom; c Department of Epidemiology and Public Health, University College London, London, United Kingdom; d Brighton and Sussex Medical School, University of Sussex Campus, Falmer, Brighton, United Kingdom

**Keywords:** Cytokines, fatigue, fMRI, insula, interoception, peripheral inflammation

## Abstract

**Background:**

Inflammation is associated with psychological, emotional, and behavioral disturbance, known as sickness behavior. Inflammatory cytokines are implicated in coordinating this central motivational reorientation accompanying peripheral immunologic responses to pathogens. Studies in rodents suggest an afferent interoceptive neural mechanism, although comparable data in humans are lacking.

**Methods:**

In a double-blind, randomized crossover study, 16 healthy male volunteers received typhoid vaccination or saline (placebo) injection in two experimental sessions. Profile of Mood State questionnaires were completed at baseline and at 2 and 3 hours. Two hours after injection, participants performed a high-demand color word Stroop task during functional magnetic resonance imaging. Blood samples were performed at baseline and immediately after scanning.

**Results:**

Typhoid but not placebo injection produced a robust inflammatory response indexed by increased circulating interleukin-6 accompanied by a significant increase in fatigue, confusion, and impaired concentration at 3 hours. Performance of the Stroop task under inflammation activated brain regions encoding representations of internal bodily state. Spatial and temporal characteristics of this response are consistent with interoceptive information flow via afferent autonomic fibers. During performance of this task, activity within interoceptive brain regions also predicted individual differences in inflammation-associated but not placebo-associated fatigue and confusion. Maintenance of cognitive performance, despite inflammation-associated fatigue, led to recruitment of additional prefrontal cortical regions.

**Conclusions:**

These findings suggest that peripheral infection selectively influences central nervous system function to generate core symptoms of sickness and reorient basic motivational states.

In healthy mammals, systemic infection triggers a set of behavioral, psychological, and physiological changes collectively known as “sickness behavior” (1,2). Symptoms include decreased motivation (e.g., fatigue, lethargy, adipsia, and anorexia), psychomotor retardation, fever, cognitive and affective change, poor concentration, confusion, depression, and impaired memory (3–5). The same stereotyped pattern of sickness behaviors is evoked across a range of infectious and inflammatory conditions, suggesting a highly coordinated reorganization of the body's physiological and motivational state to prioritize adaptive responses to pathogens and preserve bodily integrity (6). These behaviors might also be induced iatrogenically by the therapeutic administration of interferon-α (IFN-α) for the treatment of chronic viral infections and cancer (7,8). The latter observation provides direct evidence that cytokines are central to the etiology of human sickness behaviors and represents a clinical impetus for determining their underlying neurobiological basis.

Studies in rodents, using experimentally induced peripheral inflammation, emphasize a role for pro-inflammatory cytokines in mediating the production of sickness behaviors (6,9). Both early central communication of peripheral inflammatory signals (10) and subsequent motivational reorientation seem dependent upon the integrity of interoceptive vagus nerve afferents, the visceral terminals of which express cytokine binding sites (11). Early in the inflammatory response, antigen-presenting cells cluster in the vicinity of vagus nerve afferents and act as immune chemosensory elements signaling to vagal neurons via cytokine-dependent (12) and -independent mechanisms (13).

Immunohistochemical studies using the immediate early gene c-Fos to index neural activation confirm that peripheral inflammation and specifically binding of pro-inflammatory cytokines to vagus nerve receptors activate brain structures implicated in homeostasis and the representation of internal bodily state (interoception) (10). This afferent signaling is rapid; peripheral inflammation induces c-Fos expression in the primary projection nucleus of the vagus nerve (nucleus tractus solitarius [NTS]) and secondary projection regions (including parabrachial, paraventricular and supraoptic nuclei, central amygdala, and bed nucleus of the stria terminalis) within 60 min of peripheral inflammatory challenge (10). In rodents, damage to the vagus nerve attenuates specific sickness behaviors, including changes in motivation (14) and social behavior (15).

Afferent interoceptive information conveyed via the spinal cord might also contribute to central signaling of peripheral inflammation. Information traveling through spinal lamina I is predominantly tuned to motivationally salient sensations, including pain (16,17), temperature (18), itch (19), and sensual touch (20), and converges with vagus nerve afferent information within brainstem and thalamus (21). In humans, the central terminus of convergent afferent vagus and spinal interoceptive pathways within anterior insula cortex might support a consciously accessible representation of physical wellbeing (22) that is implicated as a neural substrate for subjective emotional feelings (17,22). Whether these interoceptive neural afferent pathways also provide the principle channel for rapid central signaling of peripheral inflammation in humans has not been established previously.

We combined functional magnetic resonance imaging (fMRI) with an experimental model of peripheral inflammation to address the question of how inflammatory signals are represented in the brain and to describe the neurobiological mechanisms that underlie an early shift in motivational behavior. The complementary question of how peripheral inflammation induces a change in mood and emotional processing is the focus of another publication in this journal (23). We used *Salmonella typhi* vaccination (Typhim Vi) as our inflammatory challenge. This inflammatory model has been shown previously to stimulate a low-grade peripheral inflammatory response with associated cognitive, behavioral, and emotional components of sickness (24,25). Importantly, we have also previously reported that peripheral inflammation induced with this model in this subject population does not cause a general change in the coupling between neural activity and blood-oxygen-level-dependent (BOLD) signal, the basis of inferences made in fMRI studies (26). We predicted that the central communication of peripheral inflammation is associated with pro-inflammatory cytokine release and achieved through direct modulation of interoceptive pathways engendering core cognitive and motivational components of sickness behavior, including subjective fatigue and lethargy. We used the color-word Stroop as a high-demand cognitive task that induces mental stress through marked attentional load and response-control demands. The Stroop task is frequently employed to assess high-demand cognitive processes including attentional and executive control, stimulus conflict, response monitoring, and inhibition with well described behavioral effects and neural basis (27,28). These processes are typically compromised by sickness and are a focus of the current report. We have previously reported that inflammatory influences on low-level psychomotor responses with this task are modulated via an action on the substantia nigra (26).

## Methods and Materials

### Subjects and Study Design

The study design was a randomized, double-blind crossover trial. Sixteen healthy male participants, mean age (± SD) 24.9 years (± 4.8), were tested twice a mean of 7 days apart. All were medication-free with no use of nonsteroidal or steroidal anti-inflammatory drugs in the preceding 2 weeks or any vaccination in the preceding 6 months or typhoid vaccination in the 3 years preceding study enrollment. Participants were blind to the injection order. Typhim is a standard vaccination for travel to regions with poor sanitation; other than mild sickness symptoms, local soreness, and erythema, serious reactions are rare. Procedures were approved by the joint University College London (UCL)/University College London Hospitals (UCLH) Ethics Committee. Of note, these are the same group of subjects that were reported on in our previous study (26) and in (23).

### Generation and Measurement of Inflammatory Response

Participants received injection with either .025 mg Typhim Vi (Aventis Pasteur, MSD, Maidenhead, Berkshire, United Kingdom) or .9% sodium chloride into the nondominant deltoid muscle, receiving the other injection on their return visit. Venesection was performed at baseline and 3 hours after vaccination immediately after MRI scanning. Plasma interleukin-6 (IL-6) and tumor necrosis factor α (TNF-α) were assessed with high-sensitivity two-site enzyme-linked immunosorbent assays (R&D Systems, Oxford, United Kingdom). Salivary cortisol was collected with cotton dental rolls at baseline and at 2 and 3 hours (Salivettes, Sarstedt, Leicester, United Kingdom) and analyzed with a time resolved immunoassay with fluorescence detection. Body temperature, heart rate, and resting blood pressure were measured at baseline, 2 hours, and 3 hours with sublingual digital thermometer and an electronic sphygmomanometer (A&D UA779, Tokyo, Japan), respectively.

### Behavioral and Mood Ratings

Mood and other psychological symptoms were assessed with a modified version of the Profile of Mood States (POMS) (29). This consisted of five to six items from each of five scales (vigor, fatigue, depression, tension-anxiety, and mental confusion), together with four somatic symptom items. Each was rated from 0 to 4, and scores were computed by summing ratings on individual items. Paired *t* test was used to compare responses to vaccine and placebo conditions.

### Color Word Stroop Task

The target color word was presented with the four possible response words (red yellow green blue) below. Target words and order of response words were displayed randomly. Subjects were instructed to respond as rapidly as possible to the color of the target word with a four-button response pad corresponding to the response words below. In the incongruent condition all words were printed in a color incongruent with the target word; in the congruent condition the font color of all words matched the read target word. Both targets and possible response words were displayed for 3000 msec preceded by a central fixation cross presented for 2000 msec.

### Functional Imaging and Imaging Data Analysis

Functional MRI data were acquired on a 1.5-T Siemens Sonata MR scanner (Siemens, Malvern, Pennsylvania) equipped with a standard head coil. Heart rate was continuously recorded with a pulse oximeter (Nonin 8600, Nonin Medical, Plymouth, Minnesota), probe on the left index finger. The fMRI datasets were analyzed with SPM5 (http://www.fil.ion.ucl.ac.uk/spm). The first five volumes were discarded. Individual scans were realigned and unwarped, time-corrected, normalized, and spatially smoothed with an 8-mm full-width-at-half-maximal Gaussian kernel with standard SPM methods. A high-pass frequency filter (cut off 120 sec) and corrections for auto-correlation between scans (AR1) were applied to the time series.

Each event was modeled by a standard synthetic hemodynamic response function. Congruent and incongruent trials and commission and omission errors were modeled as separate regressors in first-level multiple regression analyses. Null events were included to facilitate identification of differential hemodynamic responses to stochastically ordered stimuli. First-level design matrices for each participant were estimated within the General Linear Model. Effects of task (incongruent and congruent vs. implicit baseline) were computed on a voxel-wise basis for each participant for both vaccination and placebo conditions in the form of statistical parametric maps. Subsequent second-level paired *t* test analyses were performed on the SPM contrast images for formal inference about population effects.

Results for the main effects of task and inflammatory state were thresholded at the conservative *p* < .05 false detection rate (FDR) corrected. The main effect of inflammation was calculated as the effect of all stimulus events versus implicit baseline. Interactions and between subject correlations with fatigue, confusion, and IL-6 were thresholded at *p* < .001 uncorrected with clusters of 10 or more contiguous voxels reported to reduce risk of Type-1 error. Between subject correlations of fatigue and confusion were calculated separately for vaccine and placebo conditions with the associated Stroop activation maps (incongruent vs. congruent). Vaccine- and placebo-associated changes in diastolic and systolic blood pressure were used as co-regressors in both the analysis of the main effect of inflammation and between subject correlations. All coordinates relate to Montreal Neurological Institute space. See Supplement 1 for further details.

## Results

### Typhoid Vaccine Induces an Inflammatory Cytokine and Sickness Response

**Figure 1:**
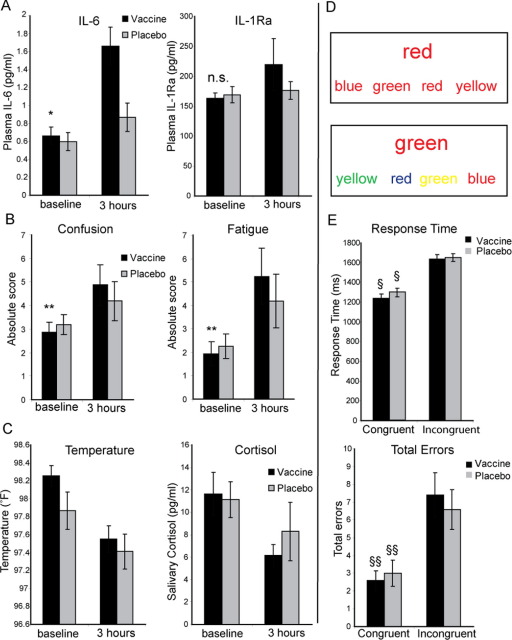
Response to inflammatory challenge. Typhoid vaccination induced a robust inflammatory cytokine response and symptoms of sickness behavior. **(A)** Plasma inflammatory cytokine level (± SEM) at baseline and 3 hours after typhoid and placebo (sodium chloride [NaCl]) injection. Interleukin-6 (IL-6) showed a significant response to typhoid but not NaCl (*p < .001; *n* = 16). There is a nonsignificant increase in IL-1Ra to typhoid and no change in tumor necrosis factor α (not shown). **(B)** Symptoms of sickness at baseline and 3 hours after typhoid and placebo. Confusion and fatigue were significantly greater after vaccine (**p < .01; *n* = 16) but not placebo. **(C)** Additional indexes of an inflammatory response. There was no significant potentially confounding increase in temperature or salivary cortisol after either vaccine or placebo (*p* > .05). **(D)** Color word Stroop task. Incongruent and congruent conditions, subjects selected the response word that correctly identified the color of the target word above. **(E)** Response time (RT) and total errors for incongruent and congruent trials in both the inflammation and placebo conditions. Response times and errors were significantly greater for incongruent trials in both vaccine and placebo conditions. ^§^Mean RT difference (± SEM) = 377.6 msec (± 21.6) [*t*(23) = 17.46, *p* < .001]. ^§§^Mean difference errors (± SEM) = 4.21 (± .66) [*t*(23) = 6.38, *p* < .001]. There was no significant main effect of inflammatory state on either RT (*p* = .31, *n* = 24) or errors (*p* = .79, *n* = 24).

After typhoid vaccination but not placebo, participants showed a marked inflammatory response with > twofold increase in plasma IL-6 from .66 ± .38 pmol/L at baseline to 1.66 ± .86 pmol/L at 3 hours (*p* < .001) (Figure 1A). The placebo condition evoked a much smaller rise in IL-6 (.60 ± .41 pmol/L at baseline to .87 ± .63 pmol/L at 3 hours, *p* = .05 (Figure 1A), consistent with a response to experimental stress (30). There was a significant treatment × sample (baseline and at 3 hours) interaction for IL-6 (*F* = 7.98, *p* = .013). Increases in plasma TNF-α or IL-1RA did not reach significance, consistent with previous findings (25). Typhoid vaccination but not placebo also elicited significant increases in subjective fatigue, confusion, and impaired concentration at 3 hours (Figure 1B). Total mood score also significantly deteriorated 3 hours after vaccine [single tailed paired *t*(15) = 1.86, *p* < .05] but not placebo [*t*(15) = 1.43, *p* > .05], as discussed in (23). As shown previously in independent populations (24), there was no effect of typhoid vaccine on other POMS subscales or ratings of illness symptoms, including fever, aching joints, nausea, and headache. Psychological changes were also unrelated to potentially confounding increases in body temperature or cortisol levels (24) (Figure 1C). See Supplement 1 for further details.

### Inflammation and Behavioral Performance on the Stroop Task

All subjects demonstrated significant increases in both response times and error rates on incongruent compared with congruent Stroop trials. This pattern, consistent with a greater cognitive cost of demanding “conflict” trials (Figure 1E), was apparent in both vaccine and placebo conditions. Of note, there were no significant differences in performance between the typhoid vaccine and placebo conditions, despite subjective reports of increased mental confusion and diminished attention associated with inflammation (although as we previously reported that there was a correlation between vaccine-induced IL-6 and across-task response time [26]). This equivalence in task performance mitigates against potential behavioral confounds in the interpretation of neuroimaging data, such that differences in neural activity can be interpreted as reflecting changes in the recruitment of cognitive resources to meet task demands rather than nonspecific changes secondary to changes in behavioral performance (Figure 1E).

#### Color Word Incongruence Activates the Fronto-Parietal Cognitive Control Network

**Figure 2:**
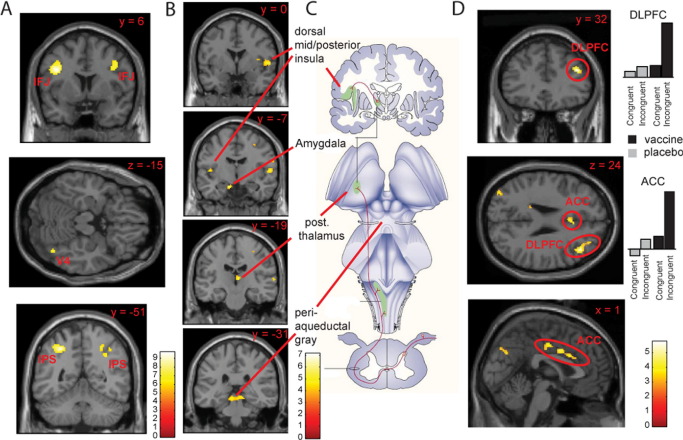
Functional magnetic resonance imaging analysis of the color word Stroop task. **(A)** Incongruent trials enhanced activity within bilateral inferior temporal junction regions, bilateral intraparietal sulci, and right V4 (plotted for illustrative purposes at false detection rate [FDR] *p* < .05 corrected). **(B)** Performance of the Stroop task (congruent and incongruent conditions combined vs. implicit baseline) under inflammation-activated projection areas of the vagus nerve and spinal lamina I afferent pathways together with the periaqueductal gray, the central autonomic efferent region (plotted at FDR *p* < .05 corrected). **(C)** Illustration of primary and secondary projection areas of the afferent vagus nerve and spinal lamina I afferents adapted by permission from MacMillan Publishers Ltd: Nature Reviews Neuroscience (17) copyright 2002. **(D)** Interaction of task and inflammatory state. Performance of cognitively demanding incongruent events under inflammation-recruited right dorsolateral prefrontal (DLPFC) and bilateral anterior cingulate cortices (ACC) (plotted for illustrative purposes at *p* < .005 uncorrected).

Cognitively demanding incongruent trials, compared with less-demanding congruent trials, evoked a robust activation (*p* < .05 FDR corrected) within regions implicated in cognitive and attentional control, including bilateral prefrontal cortex (FDR corrected *p* < .001 bilaterally) and intraparietal sulcus (FDR corrected *p* < .001 bilaterally) (31,32) (Figure 2A) (Table 2 in Supplement 1). The opposite contrast (congruent > incongruent) strongly activated two regions, orbito-medial-prefrontal-cortex (Brodmann area [BA]10) (FDR *p* < .004) and, following Vogt's nomenclature (33), ventral-posterior cingulate gyrus (vPCC) (FDR *p* < .004). This response profile accords with existing literature on their functional role. Thus, medial BA10 activity is known to inversely correlate with response times (and task difficulty) across a broad range of cognitive paradigms (34), and both medial BA10 and vPCC are part of a putative default mode network, expressing greater activity when external task demands are low (35).

### Activity Within Regions Encoding a Representation of Internal Bodily State Increases Within 3 Hours of Inflammatory Challenge

Typhoid vaccination (compared with placebo) enhanced neural activity within brainstem (FDR *p* < .017), thalamus (FDR *p* < .013), amygdale (FDR *p* < .009), cingulate (FDR *p* < .02), and bilateral mid (FDR *p* < .013 and *p* < .017, right and left, respectively) and anterior insula (FDR *p* < .028 and *p* < .025, right and left, respectively) during presentation of congruent and incongruent stimuli. All of these regions are implicated in the integration of affective and motivational processing with afferent interoceptive information, which is itself organized hierarchically across these subcortical and cortical regions (Figure 2C). However, many of these regions are also implicated in efferent autonomic control, and importantly, typhoid but not saline injection was associated with a significant increase in mean arterial blood pressure at 3 hours (2-way repeated measures analysis of variance [interaction time and inflammation *F*(2,30) = 3.58, *p* < .02].

To identify brain regions whose activity correlated specifically with afferent (interoceptive) rather than efferent autonomic responses, we therefore repeated the aforementioned analysis with the addition of co-regressors for typhoid- and saline-associated changes in blood pressure (Figure 2B) (Table 1 in Supplement 1). This analysis served to remove changes in brain activity correlating with efferent autonomic responses and corroborated our initial findings showing a main effect of inflammation in each of the aforementioned regions. It also revealed additional activations correlating with inflammation-associated changes in blood pressure within bilateral dorsal pons (encompassing parabrachial nuclei), cingulate (pMCC), and lateral right dorsal midinsula/S II (Figure 1 in Supplement 1).

### Maintenance of Cognitive Performance Under Inflammation Recruits an Extended Frontal Cortical Network

To investigate differential effects of inflammation on cognitively demanding versus less-demanding tasks, we examined the interaction between inflammation and cognitive demand. We observed more widespread engagement of activity in response to demanding incongruent trials during vaccine-induced inflammation, with recruitment of additional dorsolateral prefrontal and cingulate (MCC) cortices, compared with placebo (Figure 2D) (Table 1 in Supplement 1). Because participants overall showed no performance differences because of inflammation, these effects are attributable to the requirement of additional neural resources to maintain equivalent task-performance. Activity within both dorsolateral prefrontal and dorsal anterior cingulate cortices is typically enhanced with increasing task demands, as seen under the demands of performing a visual discrimination task in the face of cross-modal auditory conflict (36).

### Insula Activity Predicts Inflammation-Induced Fatigue

**Figure 3:**
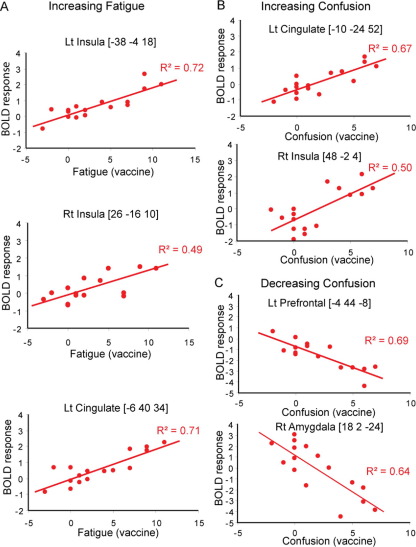
Neural regions correlating with vaccine-associated fatigue **(A)** and confusion **(B)**. Values shown are for the first eigenvariate for 8-mm diameter regions of interest centered on the coordinates shown in Table 3 in Supplement 1. Lt, left; Rt, right; BOLD, blood-oxygen-level-dependent.

Individuals showed varying susceptibility to the cognitive and motivational effects of inflammation. To explore this variability we performed a correlational analysis on our neuroimaging data to investigate interindividual differences in sensitivity to inflammation-induced fatigue and confusion, with individual contrast maps (incongruent > congruent) and participants' own reports of subjective changes after both vaccine and placebo. Inflammation-associated fatigue was predicted by activity changes within bilateral mid/posterior insula (*p* < .00002, *R*^2^ = .72, and *p* < .001, *R*^2^ = .49, right and left, respectively) and left anterior cingulate (aMCC/pACC) (*p* < .00003, *R*^2^ = .71). These activations remained after covarying out individual differences in vaccine-associated changes in diastolic and systolic blood pressure (Figure 3A) (Table 3 in Supplement 1), suggesting an association with afferent rather than efferent autonomic effects of peripheral inflammation. Importantly, fatigue after saline injection did not correlate significantly with any brain region and, in particular, did not correlate with activity in either insula or cingulate even at a liberal threshold of *p* < .01, suggesting heterogeneity in mechanisms of fatigue in the placebo condition.

Enhanced confusion was predicted by right midinsula (*p* < .001, *R*^2^ = .50) and more posterior left cingulate (dPCC) (*p* < .00006, *R*^2^ = .67) activity (Figures 3B and 3C). Again, these activations were not seen in association with confusion reported after placebo, suggesting a mechanistic role in inflammation-associated confusion. The insula regions associated with fatigue and confusion after vaccine were also activated in the “main effect” of inflammation on stimulus processing; hence our data suggest that symptomatic fatigue and mental confusion, core psychological components of sickness behavior, emerge from the interaction between efferent physiological demands of cognitive processes and bottom-up representations of internal states of wellbeing. Interestingly however, there were no significant correlations between any of the reported activations and peripheral IL-6 levels at the stringent thresholds adopted throughout the current study.

## Discussion

Studies in rodents support a central role for afferent interoceptive fibers traveling with the vagus nerve in the motivational reorientation associated with peripheral inflammation. To our knowledge this is the first study that points to a similar mechanism for sickness behavior in humans. It is known that neurons within discrete brain regions express cytokine receptors, including medial thalamic nuclei, ventromedial hypothalamus, basolateral amygdala, and cerebellar Purkinje cells (37). Therefore, one possibility is that the central neural responses we observed because of typhoid vaccination represent a direct effect of circulating cytokines on brain or even the local synthesis of cytokines as part of a systemic immune response. However, against such an interpretation is evidence that increases in cytokine levels within neural tissue after peripheral inflammatory challenge typically occur approximately 8 hours after inflammatory challenge (and have not been shown to occur within 3 hours) (38). Moreover, this central cytokine expression is modulated by earlier vagally mediated mechanisms (39,40). Instead, we suggest that interoceptive signaling provides the most parsimonious account for the observed activity change within interoceptive (and cognitive) regions.

At the conservative threshold reported here we observed inflammation-dependent activity within the right medial thalamus, encompassing the mediodorsal nucleus. This nucleus, part of the interoceptive lamina I pathway, projects to cingulate and prefrontal cortex. More lateral thalamic activity consistent with activations within the basal and posterior ventromedial nuclei (VMb and VMpo), which project to dorsal mid/posterior insula, was evident at a less stringent threshold (*p* < .001 uncorrected). Inflammation-associated activity in both bilateral dorsal-mid and anterior insula areas, however, was also seen at the more stringent threshold. The location of these activations is noteworthy. The VMb, which receives predominantly vagal fibers, and VMpo (predominantly sympathetic) project to dorsal mid/posterior insula in a rostrocaudal topographic manner, with vagal projections extending more rostrally. It is interesting therefore that our activations occur in a more rostral region of interoceptive cortex than those reported for thermal sensation (18), noxious pain, and itch (non-vagal) but close to the activation reported by Rosenkranz *et al.* (41) in response to antigen-induced inflammatory airways response in asthmatic subjects, mediated via afferents traveling with the vagus.

Direct stimulation and deactivation studies in humans (42) and animals (43), however, show that insula activity is associated with efferent autonomic change. To isolate efferent from afferent effects of inflammation, we used inflammation- and placebo-associated changes in blood pressure as co-regressors in our analysis. This confirmed that insula activity remained significant after accommodating changes in efferent autonomic activity. Blood pressure change, however, was highly correlated with dorsal pontine activity in a region encompassing the parabrachial nuclei, with much of the variance in this region after inflammation accounted for by changes in efferent autonomic activity. Interestingly, although playing a role in brainstem homeostatic reflexes including regulation of blood pressure, the parabrachial nuclei do not form part of human lamina I spinothalamocortical interoceptive pathways projecting to dorsal insula (17).

The interaction between inflammation and the enhanced cognitive demands of processing incongruent (vs. congruent) stimuli in the Stroop task permitted us to investigate the neural basis of cognitive concomitants of sickness behavior. Notably, we observed a more widespread brain engagement when processing high-demand incongruent trials with recruitment of dorsolateral prefrontal and midcingulate (aMCC/pMCC) cortices being expressed during vaccine-induced inflammation compared with the placebo condition (Figure 3D). Because participants showed no performance differences due to inflammation, these effects are most likely attributable to a requirement for additional neural resources to maintain equivalent task-performance under inflammation. Activity within both dorsolateral prefrontal and dorsal anterior cingulate cortices is typically enhanced with increasing cognitive demands. Both regions also show an increase in activity during performance of a cognitively demanding visual task in the face of cross-modal auditory distracters (36). Their concurrent activation therefore suggests that interdependent cognitive/attentional (44) and somatic (autonomic) (45) mechanisms are invoked to maintain performance and compensate for compromised fluency in cognitive processes in the face of increased conflict from interoceptive processing demands during inflammatory states.

Analysis of the neural basis of inflammation- and placebo-associated changes in fatigue and confusion also highlighted a key role for insula and cingulate cortices in mediating subjective symptoms after peripheral inflammation. Inflammation-associated but not placebo-associated fatigue was predicted by activity change within mid/posterior insula bilaterally and right anterior cingulate (pACC/aMCC). These findings suggest a degree of specificity of neural mechanisms underlying fatigue associated with peripheral inflammation that is not seen in more general placebo-associated fatigue (which might result from more heterogeneous mechanisms). Previous studies showing insula responses to graded cooling (18), itch (46), and intensity of dynamic exercise (47) support our hypothesis that inflammation-associated fatigue results from a similar insula-based interoceptive mechanism. Interestingly, this cingulate region is also activated by pain (48,49) and, in particular, pain induced by visceral more than somatosensory stimulation (50). In this context pACC/aMCC activity shows greater correlation with the reported unpleasantness than the intensity of the stimulus, suggesting a role in emotional rather than somatosensory localization components of pain (51). This role might also be supported by its strong amygdala connectivity. Its tight correlation with inflammation-associated fatigue across subjects in our present study suggests a potentially broader role for this cingulate sub-region in processing emotional components of unpleasant visceral stimuli.

Of note, there were no significant correlations between reported activations and peripheral IL-6 levels (at the stringent thresholds adopted throughout the current study) and, furthermore, no correlation between IL-6 levels and subjects' subjective symptom ratings. This finding is noteworthy, especially when compared with our previous finding of a correlation among interindividual susceptibility to inflammation-associated motor slowing, peripheral cytokine (IL-6) levels, and substantia nigra activity in this group (26). This finding suggests that, rather than reflecting a simple index of peripheral cytokine levels, subjective symptoms after peripheral inflammation likely result from a complex interplay between bottom-up processes (associated with peripheral inflammation) and top-down modulation of these processes and resultant subjective symptoms. Furthermore, although IL-6 is a useful index of inflammation and functions in an endocrine manner to coordinate inflammatory responses, it acts as part of an interacting network of pro- and anti-inflammatory cytokines. This complex relationship with other inflammatory cytokines, in particular IL-1β (which also acts on vagal afferents), suggests that a simple relationship between IL-6 levels and neural responses might be the exception rather than the rule.

To conclude, our empiric study provides a general framework for how peripheral states of inflammation (typically related to systemic infection) modulate attentional and motivational brain systems. It complements our previous study that implicated changes in substantia nigra activity in low-level psychomotor responses to inflammation (26) and our second article under consideration in this journal (23), suggesting that inflammation modulates mood via an effect on Cg25 connectivity, a region implicated in the pathogenesis of primary depression. We acknowledge that fMRI is primarily a correlative technique and that our experiment cannot prove that inflammation directly causes the pattern of activation seen and, furthermore, that our findings in a group of healthy young men might not extrapolate to other ages, gender, or health status. Also, noteworthy in this regard is the literature showing that chronic low-grade systemic inflammation, with minor increases in circulating inflammatory mediators, is associated with age-related cognitive decline (52) and dementia (53). Similarly, the therapeutic use of cytokines (in particular IL-2 and IFN-α) in the treatment of malignancies and chronic viral infections is frequently complicated by cognitive, emotional, and behavioral disturbance (54). Sickness behaviors themselves can lead to changes in immune competence by modulating host sensitivity to pathogens and influencing the degree of activation of the innate immune system and the production of peripheral cytokines. Bidirectional interactions between immune and psychological states ultimately determine the degree of wellbeing (4).

The main implication of the data we present is that they highlight a neurobiological substrate for the central representation of peripheral inflammation in humans and suggest how these representations might interact with the resource requirements of complex cognitive processing. Our findings indicate the neural mechanisms underpinning interindividual susceptibility to psychological symptoms associated with inflammation-induced sickness and have broader clinical implications across medical specialties in which these common symptomatologies might be targeted at the level of the central nervous system.
