# Supplementary material for: Neural Origins of Human Sickness in Interoceptive Responses to Inflammation
Source: Biol Psychiatry. 2009 Sep 1;66(5):415–22. doi: 10.1016/j.biopsych.2009.03.007 (PMC2885492; doi:10.1016/j.biopsych.2009.03.007)
Supplement: Supplement 1 [file mmc1.pdf]

Supplementary Materials

Materials and Methods

Subjects & study design

The study design was a randomized double-blind crossover trial. Sixteen healthy male participants (mean age (± SD) 24.9 years (± 4.8) years) were tested twice a mean of 7 days apart. All were medication free with no use of non-steroidal or steroidal anti-inflammatory drugs in the preceding two weeks, any vaccination in the preceding 6 months or Typhoid vaccination in the three years preceding enrollment in the study. All participants denied recreational drug use within the last six months. Participants were blind to the order of injection. LB and CW, who recruited and enrolled subjects, explained the task procedures, administered questionnaires, and recorded cytokine, blood pressure and cortisol measurements, were also blind to injection order. NH (a registered medical practitioner), who screened subjects, took blood samples and administered the injections of saline and Typhoid, was unblinded though played no role in the above procedures to ensure blinding of procedures while maintaining subject safety. Salmonella typhi vaccination is a standard vaccination for travel to regions with poor sanitation; other than mild sickness symptoms, local soreness and erythema, serious reactions are rare. Informed consent was obtained in accordance with the Declaration of Helsinki (Helsinki 1991) and the procedures were approved by the joint University College London (UCL/University College London Hospitals (UCLH) Ethics Committee). Participants were recruited by advertisement on the UCL campus and given a small financial reimbursement for involvement in the study. Of note these are the same group of subjects that were reported on in our previous study (1) and in (2).

Generation and measurement of inflammatory response

Participants were injected with either Salmonella typhi capsular polysaccharide vaccine 0.025 mg (Typhim Vi, Aventis Pasteur MSD, Berkshire, UK) or 0.9% NaCl into the non-dominant deltoid muscle, receiving the other injection on their return visit.

Venesection was performed at baseline and 3 hours post vaccination immediately after subjects completed MRI scanning. Participants were scanned from two to three hours after vaccination, which was designed to coincide with the previously observed peak cytokine response to Typhoid vaccination (3). Blood (10

ml) was drawn into vacutainer tubes containing EDTA as anti-coagulant, and then centrifuged immediately at 1250xg for ten minutes at room temperature. Plasma was removed, aliquoted and frozen at -70°C prior to analysis. Plasma interleukin-6 (IL-6) and tumor necrosis factor alpha (TNF-α) were assessed using high sensitivity two-site ELISAs (R&D Systems, Oxford, UK). The limit of detection of the IL-6 assay was 0.09 pg/ml, with intra-and inter-assay coefficients of variation (CVs) of 5.3 and 9.2%. The TNF-α assay had a detection limit of 0.10 pg/ml with intra- and inter-assay CVs of 6.9 and 8.4% respectively. Plasma IL-1Ra concentrations were determined by a commercial ELISA from R&D Systems (Oxford, UK). This assay had a limit of detection of 15 pg/ml and inter- and intra-assay CVs of less than 10%. Salivary cortisol was collected using cotton dental rolls at baseline, 2 and 3 hours (Salivettes, Sarstedt, Leicester, UK) and analyzed using a time resolved immunoassay with fluorescence detection. Intra- and inter-assay variability was <10% and 12% respectively. Body temperature, heart rate and resting blood pressure were measured at baseline, 2 and 3 hours using sub-lingual digital thermometer and an electronic sphygmomanometer (A&D UA779, Tokyo, Japan) respectively.

Behavioral and mood ratings

Mood and other psychological symptoms were assessed using a modified version of the Profile of Mood States (POMS) (4). This consisted of 5-6 items from each of five scales (vigor, fatigue, depression, tension-anxiety, and mental confusion), together with four somatic symptom items. Each was rated from 0-4, and scores were computed by summing ratings on individual items. Paired t-test was used to compare responses to vaccine and placebo conditions.

Color word Stroop task

The target color word was presented with the four possible response words (red yellow green blue) below. Target words and the order of response words were displayed randomly. Subjects were instructed to respond as rapidly as possible to the color of the target word using a 4 button response pad corresponding to the response words below. In the incongruent condition all words were printed in a color incongruent with the target word; in the congruent condition the font color of all words matched the read target word. Both targets and possible response words were displayed for 3000 ms preceded by a central fixation cross presented for 2000 ms. Feedback was not given. Trials were presented in 5 blocks of 36 events with 10000 ms breaks between blocks. Percentage of incongruent trials was varied between

blocks and ranged from 14% (2 blocks), 28% (1 block) to 42% (2 blocks), with 17% null events per block.

Functional imaging and imaging data analysis

Functional MRI data were acquired on a 1.5T Siemens Sonata magnetic resonance scanner equipped with a standard head coil. Mild external head restraint was used to minimize head movement during scanning. Heart rate was continuously recorded using a pulse oximeter (Nonin 8600, Nonin Medical Inc., Plymouth, MN), pulse probe on the left index finger. Visual stimuli were projected onto a screen visible via a mirror on the head coil.

Functional brain imaging data were acquired using T2*-weighted echoplanar imaging, sensitive to BOLD contrast. For the Stroop task, data were acquired with whole brain coverage using a sequence with 44 contiguous slices, 2mm slice thick, 1mm inter-slice gap, tilted -30˚ from intercommisural plane, TE 40 ms, TR 3.96 s per volume (5). 248 volumes were acquired for each participant in a single session of 16.4 minutes.

Functional MRI datasets were analyzed using SPM5 (http://www.fil.ion.ucl.ac.uk/spm). The first five volumes were discarded to allow for T1 equilibration effects. Individual scans were realigned and unwarped, time-corrected, normalized and spatially smoothed with an 8-mm FWHM Gaussian kernel using standard SPM methods. A high-pass frequency filter (cut off 120 s) and corrections for auto-correlation between scans (AR1) were applied to the time series.

In analysis of the Stroop task, each event was modeled by a standard synthetic hemodynamic response function at each voxel across the whole brain. Congruent and incongruent trials and errors of commission (response within 3 seconds) and omission (response after 3 seconds) errors were modeled as separate regressors in first-level multiple regression analysis. In the experimental design (see above) null events were included to facilitate identification of differential hemodynamic responses to stochastically-ordered stimuli.

The first level individualized design matrices for each participant were estimated within the General Linear Model. Effects of task (incongruent and congruent versus an implicit baseline) were computed on a voxel-wise basis for each participant for both vaccination and placebo conditions in the form of statistical parametric maps (SPMs) of discrete contrasts. Subsequent second-level paired t-test analyses were performed on the SPM contrast images for formal inference about population effects. Results for the main effects of task and inflammatory state were thresholded at the conservative $p < 0.05$ false detection rate (FDR) corrected with

only clusters of 10 or more voxels reported. The main effect of inflammation was calculated as the effect of all stimulus events (incongruent and congruent) vs. an implicit baseline. Interactions and between subject correlations with fatigue, confusion and IL-6 were thresholded at $p < 0.001$ uncorrected with only clusters of 10 or more contiguous voxels reported to reduce the risk of Type-1 error. Between subject correlations of fatigue and confusion were calculated separately for the vaccine and placebo conditions using the associated Stroop activation maps (incongruent vs. congruent stimuli). Vaccine and placebo associated changes in diastolic and systolic blood pressure (calculated as the difference between baseline blood pressure and average blood pressure between 2 and 3 hours post injection) were used as co-regressors in both the analysis of the main effect of inflammation and the between subject correlations. All co-ordinates relate to MNI space.

Results

Change in inflammatory cytokines following vaccine and placebo

Vaccine		Placebo
IL-6	$t(15) = 4.80, p < 0.001$	$t(15) = 2.13, p = 0.05$
IL-1Ra	$t(15) = 1.36, p = 0.19$	$t(15) = 1.74, p = 0.10$
TNF-α	$t(15) = 0.10, p = 0.93$	$t(15) = 0.06, p = 0.95$

Changes in other physical measures following vaccine and placebo

Temperature

Vaccine	Placebo
Baseline: mean(± SE) = 36.8 (±0.06) ºC	mean(± SE) = 36.6 (±0.12) ºC
2 hours: mean(± SE) = 36.7 (±0.07) ºC	mean(± SE) = 36.5 (±0.10) ºC
3 hours: mean(± SE) = 36.4 (±0.08) ºC	mean(± SE) = 36.5 (±0.10) ºC

No significant increase in temperature following vaccine or placebo $p > 0.05$.

Cortisol

Vaccine	Placebo
Baseline: mean(± SE)= 11.65 (±1.92) µg/ml	mean(± SE)= 11.12 (±1.59) µg/ml
2 hours: mean(± SE)= 7.48 (±1.45) µg/ml	mean(± SE)= 8.36 (±1.69) µg/ml
3 hours: mean(± SE)= 6.02 (±1.01) µg/ml	mean(± SE)= 8.28 (±2.61) µg/ml

No significant increase in cortisol following either vaccine or placebo $p > 0.05$.

Change in subjective sickness ratings following vaccine & placebo (POMS)

TOTAL MOOD

Vaccine, $F(2,15) = 3.263$, $\boldsymbol{p < 0.05}^*$ **Placebo**, $F(2,15) = 2.275$, $p = 0.12$

SOMATIC

Vaccine, $F(2,15) = 1.552$, $p = 0.23$ **Placebo**, $F(2,30) = 2.97$, $p = 0.09$

POMS Subscales

1) Tension

Vaccine, $F(2,15) = 1.99$, $p = 0.17$** **Placebo,** $F(2,30) = 0.08$, $p = 0.83$**

2) Depression

Vaccine, $F(2,30) = 0.91$, $p = 0.39$** **Placebo,** $F(2,30) = 1.53$, $p = 0.246$**

3) Fatigue

Vaccine, $F(2,15) = 9.239$, $\boldsymbol{p = 0.003}$*** **Placebo,** $F(2,15) = 3.090$, $p = 0.08$

4) Confusion

Vaccine, $F(2,15) = 7.636$, $\boldsymbol{p = 0.002}$ [$] **Placebo**, $F(2,15) = 1.931$, $p = 0.18$**

5) Vigor

Vaccine, $F(2,15) = 4.265$, $\boldsymbol{p = 0.04}$ [$$] **Placebo,** $F(2,15) = 5.921$, $\boldsymbol{p = 0.01}$ [$$$]

* driven by change between 2 and 3 hours $F(1) = 4.49$, $\boldsymbol{p < 0.05}$.

** significance reported after Greenhouse-Geisser correction of the degrees of freedom (Mauchly's $W < 0.05$).

*** driven by change between 2 and 3 hours $F(1) = 11.50$, $\boldsymbol{p = 0.004}$.

[$] driven by change between 2 and 3 hours $F(1) = 10.20$, $\boldsymbol{p = 0.006}$.

[$$] driven by change between 2 and 3 hours $F(1) = 12.17$, $\boldsymbol{p = 0.003}$.

[$$$] driven by change between 2 and 3 hours $F(1) = 10.48$, $\boldsymbol{p = 0.006}$ & between 2 hours and baseline $F(1) = 3.28$, $\boldsymbol{p = 0.03}$.

Supplementary Table 1: Main effect of inflammation & interaction of inflammation and task

A) Brain regions showing greater activation to both congruent and incongruent tasks under inflammation (main effect of inflammation). Results are reported for clusters of ten or more contiguous voxels after whole brain false detection rate (FDR) correction at $p < 0.05$. Vaccine and placebo associated changes in diastolic and systolic blood pressure were used as co-regressors. No region showed greater activation following placebo at this threshold. **B**) Interaction of task (congruent vs. incongruent trials) and inflammation (inflammation vs. placebo). Gray matter regions showed greater activation to congruent or incongruent trials under inflammation. Significant clusters are reported at an extent threshold of ten or more contiguous voxels surpassing a magnitude threshold of $p < 0.001$ uncorrected.

Side	Region		*MNI*		*Z*	*Cluster*	*p*
**A) Main effect vaccine>placebo**		*x*	*y*	*z*	*score*	*size*	*value*
R	*Inferior temporal gyrus*	*52*	*-60*	*-14*	*5.37*	*44*	*FDR < 0.002*
R	*Postcentral gyrus*	*62*	*-12*	*22*	*5.33*	*50*	*FDR < 0.002*
L	*Amygdala*	*-12*	*-8*	*-20*	*4.73*	*35*	*FDR <0.009*
R	*Insula (dorsal middle)*	*54*	*-2*	*4*	*4.59*	*161*	*FDR < 0.013*
L	*Insula (dorsal middle/posterior)*	*-46*	*-6*	*8*	*4.30*	*38*	*FDR < 0.017*
L	*Insula (anterior)*	*-36*	*18*	*4*	*4.00*	*33*	*FDR < 0.025*
R	*Insula (anterior)*	*34*	*10*	*6*	*3.89*	*48*	*FDR < 0.028*
R	*Thalamus*	*6*	*-20*	*8*	*4.52*	*34*	*FDR < 0.013*

R	*Periaqueductal gray matter*	*-6*	*-34*	*-10*	*4.34*	*168*	*FDR <0.017*
R	*Cingulate (pMCC)*	*6*	*-14*	*56*	*4.20*	*31*	*FDR <0.020*
**Main effect placebo>vaccine**							
	Nil						
**B) Interaction (increased activation to incongruence during inflammation)**							
R	*Dorsolateral prefrontal cortex*	*42*	*32*	*28*	*4.12*	*49*	*< 0.001*
L	*Insula (dorsal posterior)*	*-38*	*-30*	*22*	*4.04*	*52*	*< 0.001*
L	*Cingulate (aMCC/pMCC)*	*-2*	*2*	*38*	*3.70*	*15*	*< 0.001*
R	*Cingulate (aMCC)*	*6*	*22*	*26*	*3.66*	*20*	*< 0.001*
R	*Cingulate (pMCC)*	*8*	*-14*	*44*	*3.60*	*33*	*< 0.001*
R	*Precuneus BA 7*	*10*	*-54*	*42*	*3.56*	*11*	*< 0.001*
**Increased activation to congruence during inflammation**							
R	*Parahippocampal/ cerebellum*	*20*	*-36*	*-26*	*3.84*	*36*	*< 0.001*
L	*Inferior temporal gyrus*	*-48*	*-24*	*-14*	*3.55*	*20*	*< 0.001*

Supplementary Table 2: Main effect of performing the color word Stroop task. Results reported for clusters of ten or more contiguous voxels after whole brain false detection rate (FDR) correction at $p < 0.05$.

Side	Region		*MNI*		*Z*	*Cluster*	*p*
**Incongruent>Congruent**		*x*	*y*	*z*	*score*	*size*	*value*
L	*Inferior frontal gyrus (DLPFC)BA 6/9/44*	*-40*	*8*	*30*	*6.53*	*297*	*FDR < 0.001*
R	*Inferior frontal sulcus(DLPFC)BA 6/9/44*	*42*	*6*	*40*	*5.51*	*116*	*FDR < 0.001*
L	*Intra-parietal sulcus BA40*	*-28*	*-54*	*46*	*6.39*	*541*	*FDR < 0.001*
R		*30*	*-48*	*42*	*5.76*	*141*	*FDR < 0.001*
R	*Dorsolateral prefrontal cortex (BA46)*	*48*	*34*	*20*	*6.10*	*253*	*FDR < 0.001*
R	*Precuneus BA 7*	*4*	*-66*	*50*	*5.79*	*25*	*FDR < 0.001*
L		*-6*	*-68*	*52*	*5.42*	*24*	*FDR < 0.003*
R	*Superior parietal lobule BA 19*	*22*	*-76*	*40*	*5.49*	*55*	*FDR < 0.002*
L	*Middle frontal gyrus (Premotor) BA 6*	*-24*	*0*	*44*	*5.44*	*16*	*FDR < 0.003*
R	*Fusiform gyrus V4*	*40*	*-66*	*-18*	*5.32*	*13*	*FDR < 0.005*
**Congruent>Incongruent**							
L	*Posterior cingulate gyrus (vPCC)*	*-8*	*-54*	*14*	*5.11*	*91*	*FDR < 0.004*
L	*Orbito-medial prefrontal*	*-4*	*52*	*-6*	*4.92*	*205*	*FDR < 0.004*

Supplementary Table 3: Regions correlating with vaccine and placebo associated fatigue and confusion.

Results reported for clusters of greater than ten contiguous voxels at $p < 0.001$ uncorrected. Changes in systolic and diastolic blood pressure 3 hours after vaccine and placebo compared to baseline were used as regressors of no interest. Areas shown with a * were also significantly activated as a main effect of vaccination (inclusive mask at $p < 0.05$ uncorrected). Placebo associated fatigue did not positively correlate with any region at this threshold and in particular with any insula or cingulate region even at a highly conservative threshold of $p < 0.01$. R^2 values shown are for the first eigenvariate of an 8 mm diameter spherical region of interest centered on the peak voxel coordinates shown.

Side	Region		*MNI*		*Z*	*Cluster*	*p*	
**Correlation with increasing fatigue (vaccine)**		*x*	*y*	*z*	*Score*	*Size*	*value*	R^2
L	*Insula (middle)**	*-38*	*-4*	*18*	*4.44*	*59*	*0.00002*	*0.72*
R	*Insula (middle/posterior)**	*28*	*-16*	*10*	*4.52*	*69*	*0.001*	*0.49*
L	*Cingulate (aMCC/pACC)*	*-6*	*40*	*34*	*3.85*	*17*	*0.00003*	*0.71*
**No correlation with increasing fatigue (placebo)**								
**Correlation with increasing confusion (vaccine)**								
L	*Cingulate (dPCC)*	*-10*	*-24*	*52*	*3.78*	*15*	*0.00006*	*0.67*
R	*Insula (middle)**	*48*	*-2*	*4*	*3.95*	*36*	*0.001*	*0.50*
**Correlation with increasing confusion (placebo)**								
L	*Extrastriate visual cortex*	*-4*	*-86*	*18*	*3.55*	*11*	*0.0003*	*0.59*

**Negative correlation with fatigue (vaccine)**								
R	*Middle temporal gyrus*	*46*	*10*	*-28*	*3.48*	*21*	*0.00003*	*0.71*
L	*Middle temporal gyrus*	*-46*	*14*	*-22*	*3.91*	*42*	*0.0009*	*0.53*
**Negative correlation with fatigue (placebo)**								
L	*Middle temporal gyrus*	*62*	*-14*	*-24*	*3.53*	*16*	*0.006*	*0.39*
**Negative correlation with confusion (vaccine)**								
L	*Orbito-medial prefrontal**	*-4*	*44*	*-8*	*3.81*	*70*	*0.00004*	*0.69*
R	*Amygdala*	*18*	*2*	*-24*	*3.81*	*46*	*0.0002*	*0.64*
R	*Middle temporal gyrus**	*50*	*-8*	*-22*	*4.02*	*109*	*0.0001*	*0.65*
**Negative correlation with confusion (placebo)**								
R	*Middle temporal gyrus*	*62*	*-16*	*-22*	*4.09*	*28*	*0.0001*	*0.64*

* Also activated as a main effect of vaccination (mask of vaccine > placebo thresholded at $p < 0.05$ uncorrected).

Supplementary Figure 1: Pontine activations associated with MAP change

Correlation with inflammation associated mean arterial blood pressure (MAP) change (contrast MAP change vaccine > MAP change placebo) in bilateral pons encompassing parabrachial nuclei.

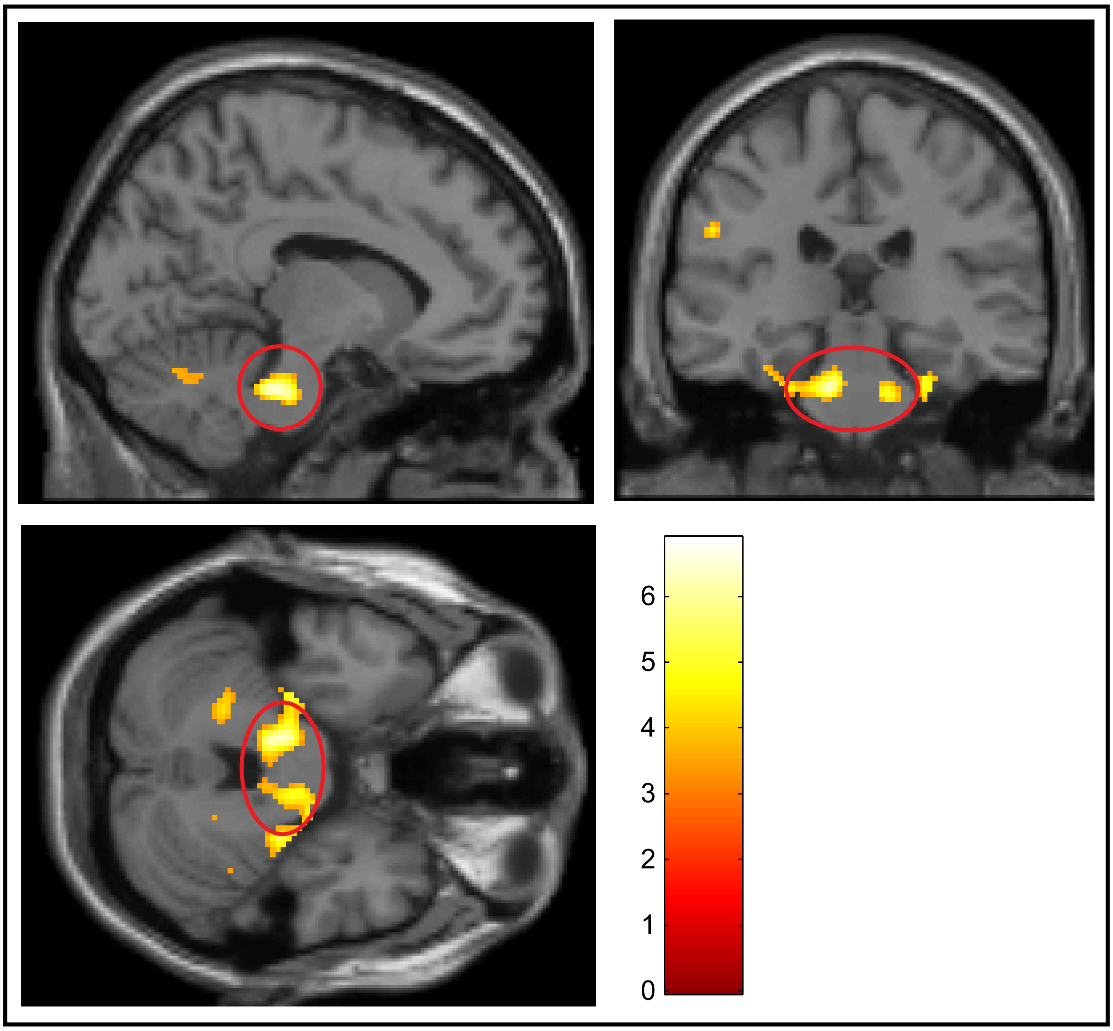

References

1. Brydon L, Harrison NA, Walker C, Steptoe A, Critchley HD (2008): Peripheral inflammation is associated with altered substantia nigra activity and psychomotor slowing in humans. *Biol Psychiatry* 63: 1022-1029.

2. Harrison NA, Brydon L, Walker C, Gray MA, Steptoe A, Critchley HD (2008): Inflammation causes mood change through alterations in subgenual cingulate activity and mesolimbic connectivity. *Biol Psychiatry* (in Press).

3. Strike PC, Wardle J, Steptoe A (2004): Mild acute inflammatory stimulation induces transient negative mood. *J Psychosom Res* 57: 189-194.

4. McNair DM, Lorr N, Droppleman LF (1981): *Manual for the profile of mood states*. San Diego (CA): Education and Industrial Testing Service.

5. Deichmann R, Gottfried JA, Hutton C, Turner R (2003): Optimized EPI for fMRI studies of the orbitofrontal cortex. *Neuroimage* 19: 430-441.
